# BMP-treated human embryonic stem cells transcriptionally resemble amnion cells in the monkey embryo

**DOI:** 10.1242/bio.058617

**Published:** 2021-09-22

**Authors:** Sapna Chhabra, Aryeh Warmflash

**Affiliations:** 1 Systems Synthetic and Physical Biology graduate program, Rice University, Houston, TX 77005, USA; 2 Department of Biosciences, Rice University, Houston, TX 77005, USA; 3 Department of Bioengineering, Rice University, Houston, TX 77005, USA

**Keywords:** Amnion, Differentiation, Human embryonic stem cells, ScRNA-seq, Trophectoderm, BMP4, Extra-embryonic mesoder

## Abstract

Human embryonic stem cells (hESCs) possess an immense potential to generate clinically relevant cell types and unveil mechanisms underlying early human development. However, using hESCs for discovery or translation requires accurately identifying differentiated cell types through comparison with their *in vivo* counterparts. Here, we set out to determine the identity of much debated BMP-treated hESCs by comparing their transcriptome to recently published single cell transcriptomic data from early human embryos ( Xiang et al., 2020). Our analyses reveal several discrepancies in the published human embryo dataset, including misclassification of putative amnion, intermediate and inner cell mass cells. These misclassifications primarily resulted from similarities in pseudogene expression, highlighting the need to carefully consider gene lists when making comparisons between cell types. In the absence of a relevant human dataset, we utilized the recently published single cell transcriptome of the early post implantation monkey embryo to discern the identity of BMP-treated hESCs. Our results suggest that BMP-treated hESCs are transcriptionally more similar to amnion cells than trophectoderm cells in the monkey embryo. Together with prior studies, this result indicates that hESCs possess a unique ability to form mature trophectoderm subtypes via an amnion-like transcriptional state.

This article has an associated First Person interview with the first author of the paper.

## Abstract (precis)

**Summary:** We show that BMP-treated human embryonic stem cells (hESCs) are more likely to represent an amnion rather than a trophectoderm cell type.

## INTRODUCTION

Human embryonic stem cells (hESCs) provide a unique window into early stages of human development. Over the last few years, they have been used to generate many medically relevant cell types and models of early human development (Dupont et al., 2019; Fu et al., 2021). However, lacking the spatial context that the embryo provides, the *in vivo* identity of cells obtained from differentiating hESCs is often unclear. The identity of BMP-treated hESCs has been particularly controversial, with arguments made for three different extra-embryonic tissues – trophectoderm, amnion and extra-embryonic mesoderm (Xu et al., 2002; Bernardo et al., 2011; Shao et al., 2017a,b). Based on limited transcriptional data from the human and monkey embryo, we previously argued that BMP-treated hESCs are more likely to represent trophectoderm cells than extra-embryonic mesoderm cells (Chhabra et al., 2019). However, it was not possible to make a direct comparison with human amnion cells due to the lack of *in vivo* data.

Obtaining data directly from human embryos is of paramount importance because there are significant differences between human embryos and those of mammalian model organisms such as the mouse, especially in the formation of amnion – the extra-embryonic tissue that covers the embryo in a protective sac (Dobreva et al., 2010; Rossant et al., 2015). In human and monkey embryos, the amnion is formed prior to gastrulation, whereas in mouse it is formed after gastrulation and is partially derived from primitive streak cells (Kinder et al., 1999; Dobreva et al., 2010). There have been no reports on the molecular characterization or lineage relationships of the amnion in humans until recently.

In a major breakthrough, a recent study (Xiang et al., 2020) succeeded in obtaining the transcriptional signature of cultured human embryos in the second week of embryonic development (Xiang et al., 2020). This study provided transcriptomes for all major cell types in the human embryo from embryonic day 6 to 14 (D6–D14) and included the first transcriptomes of putative amnion cells (AME cells – 2 cells at D12 and 11 cells at D14).

To discern the *in vivo* identity of BMP-treated hESCs, we first reexamined whether the data in Xiang et al. support labeling the cells denoted as amnion as a distinct cell type as prior studies have hinted at a transcriptional similarity between amnion and trophectoderm cells. Monkey amniotic cells *in vivo* or purported human amnion cells *in vitro* express TFAP2A, GATA2/3, CDX2, and TP63, all well-known trophectoderm markers (Sasaki et al., 2016; Shao et al., 2017a,b; Knöfler et al., 2019). Surprisingly, Xiang et al. neither examined the transcriptional similarity of the two fates nor provided a rationale for assignment of amnion fate to cells.

Our analyses revealed that cells labelled as amnion comprise a mix of different cell types, most of which are indistinguishable from syncytiotrophoblast cells. The mislabeling in the Xiang et al. study can be attributed to the inclusion of pseudogenes in those analyses. In the absence of a molecular signature for the human amnion, we turned to the recently published monkey embryo single cell transcriptome (Ma et al., 2019) to resolve the identity of BMP-treated hESCs. Comparing the transcriptional signature of BMP-treated hESCs with early post-implantation monkey amnion and trophectoderm cells revealed that they are more similar to monkey amnion cells. Together with prior studies that have revealed the functional similarity of BMP-treated hESCs with human trophectoderm cells (Xu et al., 2002; Li et al., 2013), this result potentially hints at an ability of hESCs to differentiate into trophectoderm cells through an intermediate amnion-like transcriptional state. Our analyses also revealed additional mislabeled cellular populations in the Xiang et al. dataset. Notably, the cells identified as a novel intermediate cell type likely represent extra-embryonic mesodermal cells, a transient extra-embryonic cell population that also develops prior to gastrulation in the human and monkey embryo (Luckett, 1978; Enders and King, 1988; Kinder et al., 1999). Additionally, putative inner cell mass cells are likely mislabeled cytotrophoblast cells. In summary, our analysis reveals the transcriptional similarity of BMP-treated hESCs with early post implantation monkey amnion, provides a corrected dataset based on the work of Xiang et al. that can be used to study early human development, and suggests that more work will be needed to identify the *in vivo* transcriptome of human amnion.

## RESULTS

### Notation

In the analyses that follow, we utilized the previously published single cell transcriptome data of the human and monkey embryo (Ma et al., 2019; Xiang et al., 2020) and the bulk transcriptome data of BMP-treated hESCs (Chhabra et al., 2019), which are represented with a distinct symbol – a monkey, a human and cells, respectively. If a figure contains data from multiple datasets, the relevant symbols are placed next to each plot. Otherwise, for figures where only one dataset is used, the identity of the dataset is indicated by a symbol placed next to the first panel in the figure. For simplicity, we continue to refer to mislabeled cell types by the name given in the original paper but add an apostrophe to indicate that this label is incorrect. Thus we represent the cell types from the Xiang et al. human embryo dataset (Xiang et al., 2020) as: ‘AME for putative amniotic epithelium, ‘Intermediate for the putative novel intermediate population and ‘ICM for the putative inner cell mass cells.

### ‘AME express trophoblast specific lineage genes

Although primate amnion is presumably derived from epiblast cells (Dobreva et al., 2010), both monkey amnion cells and hESC derived putative amnion cells exhibit transcriptional similarity with trophectoderm cells (Sasaki et al., 2016; Shao et al., 2017a,b; Knöfler et al., 2019). To determine the similarity of amnion with epiblast and trophectoderm lineages, we compared the expression of lineage specific genes between individual ‘AME cells in the Xiang et al. dataset with the average expression of these genes in cells corresponding to the three lineages – the epiblast, primitive endoderm and the trophectoderm.

We utilized the lineage-specific genes documented in the Stirparo et al. (2018) study, which consolidated data from previous studies (Chen et al., 2009; Roode et al., 2012; Niakan and Eggan, 2013; Blakeley et al., 2015; Deglincerti et al., 2016; Petropoulos et al., 2016; Shahbazi et al., 2016) and identified a group of 12 high confidence lineage specific genes – NANOG, SOX2, KLF17, TDGF1, PDGFRA, GATA6, GATA4, SOX17, GATA3, GATA2, KRT18, TEAD3 that effectively separate the three lineages of the pre-implantation human embryo (Stirparo et al., 2018). We replaced KRT18 with another well-known trophectoderm marker KRT7 (Shahbazi et al., 2016), as the latter was more specific to trophectoderm lineages in pre and peri-implantation stage embryos in the Xiang et al. (2020) dataset (Fig. S1D).

This known lineage marker gene set effectively separates the three lineages, even at the post-implantation stage, in both principal component and correlation analyses (Fig. S1C,E,F). It also correctly placed the derived cell types with their respective parent lineages – syncytiotrophoblast (STB) and extra-villous cytotrophoblast (EVT) cells with cytotrophoblast cells (CTB) and primitive streak cells with epiblast cells.

**Fig. 1.**
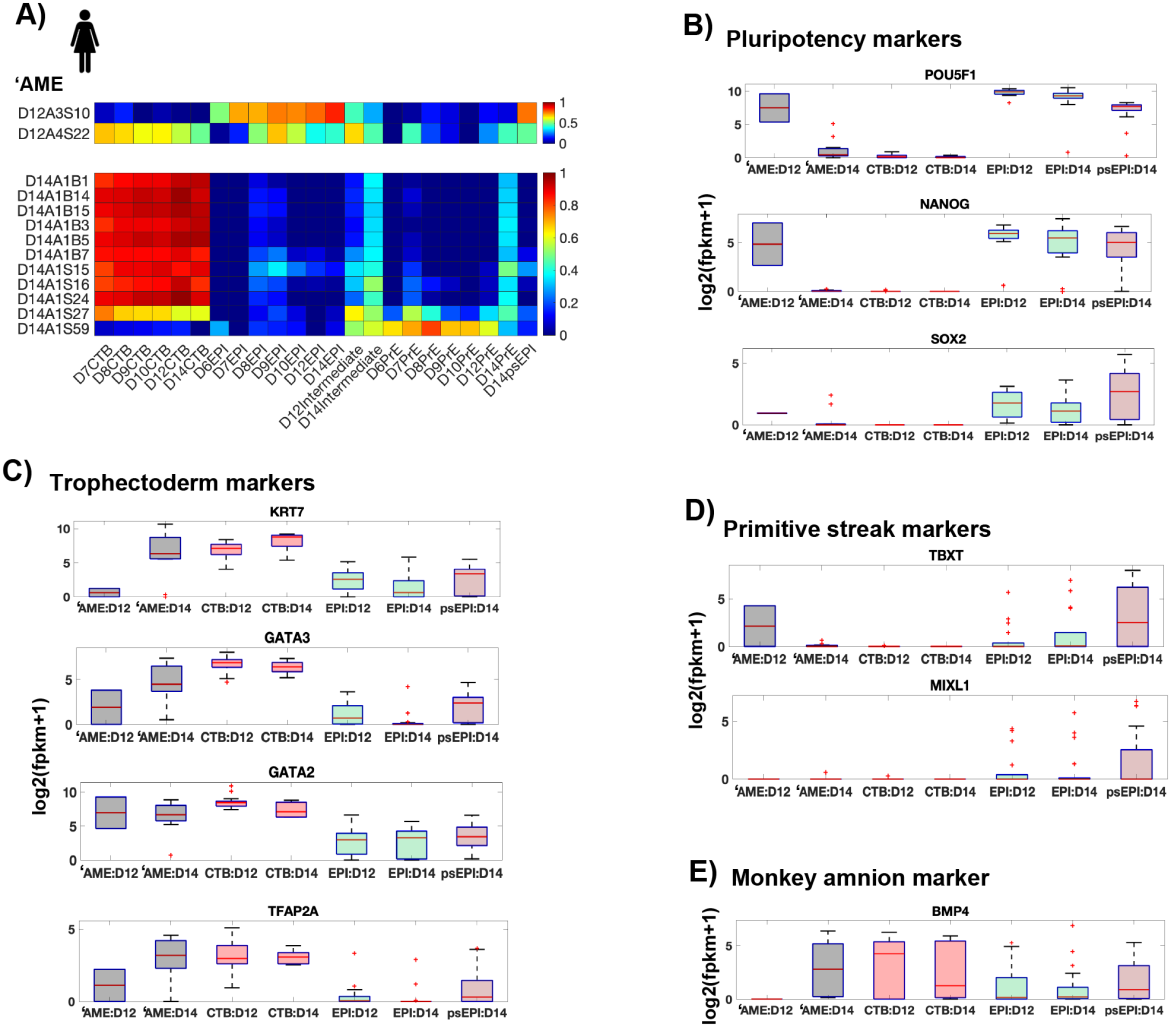
**‘AME cells express trophoblast lineage specific genes.** (A) Heatmap showing Pearson correlation coefficients for expression of known lineage markers corresponding to human trophectoderm, epiblast and primitive endoderm lineages (12 genes represented in Fig. S1C), between individual ‘AME cells and average expression of same genes in other indicated cell types. (B–E) Box plots showing expression of indicated genes in indicated cell types.

**Fig. 2.**
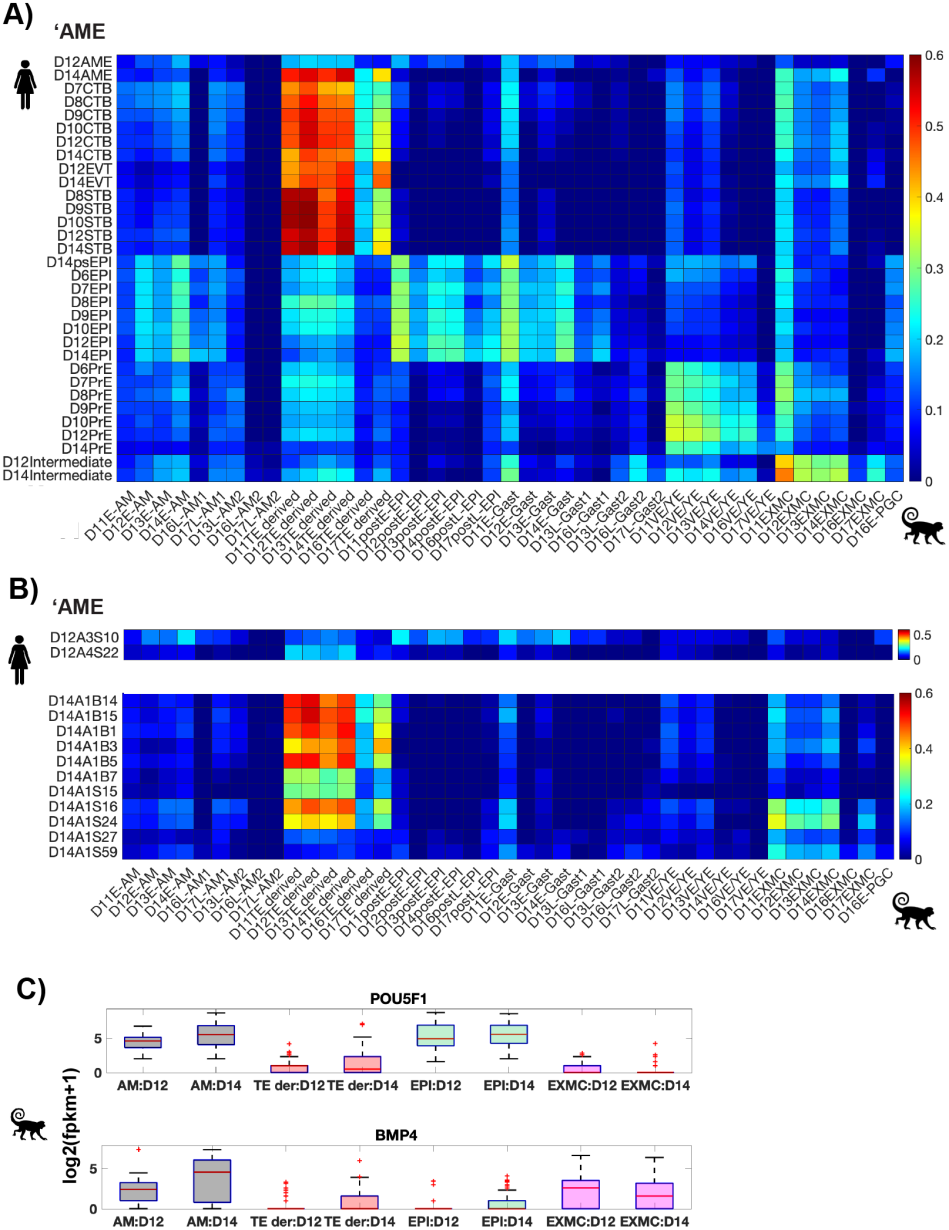
**‘AME cells are transcriptionally more similar to monkey trophectoderm-derived than monkey amnion cells.** (A) Heatmap showing Pearson correlation coefficients of average expression of variable genes in the monkey embryo [(C,E); CV>1 across 1453 monkey cells; 2440 genes] in indicated cell types. (B) Heatmap showing Pearson correlation coefficients for expression of variable genes in the monkey embryo between individual human AME cells and monkey cell type averages. Genes used in A and B are the same as those in Fig. 2D. (C) Box plots showing expression of indicated genes in indicated lineages. The symbols represent species corresponding to the two datasets (human and monkey).

In tSNE analyses presented in Xiang et al. (2020), D12 ‘AME cells are placed with epiblast cells, while the D14 ‘AME cells are placed in the PSA (primitive streak anlage) cluster, indicating transcriptional similarity of amnion with epiblast and primitive streak cells (Xiang et al., 2020; Figs 1A and 2). However, our analyses with lineage specific genes contradicts this result and instead shows transcriptional similarity of D14 amnion cells with trophectoderm cells, not with epiblast or primitive streak cells (Fig. 1E). Consistent with this, most D14 ‘AME cells do not express known pluripotency and primitive streak markers, thus questioning their placement in the PSA cluster (Fig. 1B,D).

Most amnion cells (11/13; 1/2 D12, 10/11 D14) are transcriptionally correlated with CTB cells (Fig. 1A). Consistent with this, most D14 amnion cells express trophectoderm markers – KRT7, GATA2/3, TFAP2A at levels comparable to D14 CTB cells (Fig. 1C). Strikingly, although the ‘AME express high levels of KRT7 in the scRNA seq data, only the trophectoderm but not the amnion, was positive for KRT7 in immunostaining in the same study (Xiang et al., 2020; Fig. 1J, CK7/KRT7 stain). This suggests that the cells labelled as ‘AME either post-transcriptionally repress KRT7 or represent mislabeled CTB/CTB-derived cells.

### ‘AME are transcriptionally more similar to monkey trophectoderm-derived cells than monkey amnion cells

To further discern the identity of ‘AME, we compared the transcriptomes of cells in the human embryo in the Xiang et al. study with cells in the post-implantation cynomolgus monkey embryo (Ma et al., 2019), which has a very similar morphology as that of the human embryo in the peri-implantation stages (Luckett, 1978). Remarkably, known lineage markers in human embryo also delineate the three lineages in the monkey embryo, highlighting conserved expression of these genes across the two species (Fig. 2A–C).

In the known lineage gene space, most monkey cell types exhibit transcriptional similarity with their parent or sibling lineages. Amnion and gastrulating cells (primitive streak cells) are transcriptionally similar to epiblast cells, which is their parent lineage (Fig. S2C). Most amnion cells, however, are also transcriptionally similar to trophectoderm-derived cells, consistent with the expression of trophectoderm-specific genes in the monkey amnion (Sasaki et al., 2016). Extra-embryonic mesodermal cells (EXMC), which overlay the amnion and develop prior to primitive streak formation in primates (Luckett, 1978; Enders and King, 1988), are transcriptionally similar to visceral/yolk sac endoderm (VE/YE), consistent with these cells being primitive endoderm derivatives (Nakamura et al., 2016).

As this restricted lineage gene space does not distinguish the amnion and trophectoderm lineages, we repeated the analysis considering genes with variable expression across all monkey cells (CV >1, 1453 cells; 2440 genes). In this space, the amnion cells retain transcriptional similarity with the epiblast but lose similarity with the trophectoderm (Fig. S2D). Thus, expression of these genes can be utilized to determine whether human ‘AME represent epiblast derived amnion cells, as suggested by Xiang et al. (2020), or mislabeled trophectoderm cells, as suggested in the previous section (Fig. 1A-E,F).

Comparing the expression of genes with variable expression in the monkey embryo with mean expression of same genes in the human embryo reveals that human ‘AME are transcriptionally most correlated with monkey trophectoderm-derived cells, not with monkey amnion, further challenging the identities assigned to these cells in Xiang et al. (2020) (Fig. 2A). This correlation is retained at the level of single ‘AME cells, where most ‘AME cells (10/13; 1/2 D12, 9/11 D14) show highest correlation with monkey trophectoderm cells (Fig. 2B). Consistent with this, ‘AME express trophectoderm genes as shown previously (Fig. 1C).

Notably, ‘AME do not exhibit the BMP4+/POU5F1+ (OCT4+) transcriptional signature of the monkey amnion (Fig. 2C; Sasaki et al., 2016; Ma et al., 2019). D12 ‘AME do not express BMP4. D14 ‘AME express BMP4 comparable to D14 CTB cells and POU5F1 less than D14 epiblast cells (Fig. 1E,B). This is contrary to their monkey counterparts, which express BMP4 higher than trophectoderm-derived cells and POU5F1 comparable to epiblast cells (Fig. 2C).

To sum, the transcriptional similarity of ‘AME with monkey trophectoderm-derived cells and not monkey amnion cells, supports the notion that they represent mislabeled trophectoderm cells.

### Pseudogenes leads to the misclassification of ‘AME

**Fig. 3.**
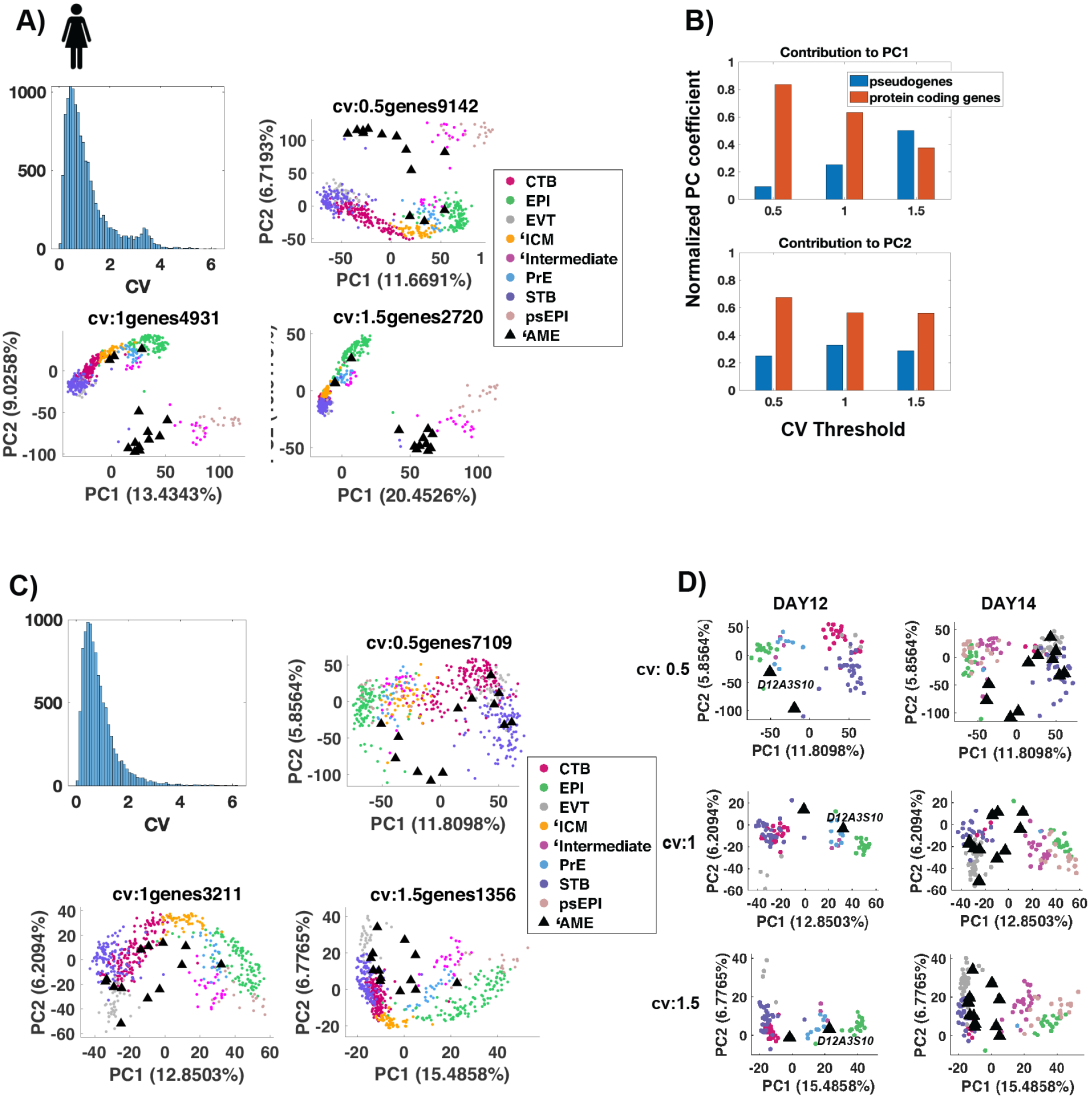
**Pseudogenes lead to placement of ‘AME cells in the PSA cluster.** (A,C) Histogram of coefficient of variation of expressed genes (genes with FPKM>1 in at least 50% cells of a given lineage) across all cells. Principal component analyses (PCA) of 555 cells with variable expressed genes. Variability is defined by a threshold in CV values. The threshold CV and the number of genes that cross the threshold are indicated above the graph. The percentage in x/y labels represents the % of variance in the data explained by each PC. Read counts calculated as log2(fpkm+1) were used for the PCA. Cells are color-coded by the lineages assigned in Xiang et al. (2020). In C, the genes were filtered to include only protein coding genes, prior to determining variable expressed genes. (B) Contribution of different gene biotypes to the first two principal components. Normalized PC coefficient=cumulative PC coefficient for a biotype/cumulative PC coefficient for all biotypes. Cumulative PC coefficient is calculated as the sum of the absolute PC1 coefficient of all genes in that biotype. The two biotypes that contribute the most are shown. (D) PCA plots in C plotted for a subset of cells corresponding to embryonic day 12 and 14.

We next sought to understand the reason that the analyses in Xiang et al. (2020) mistakenly classified human ‘AME cells as amnion, rather than trophectoderm. To delineate lineages in the human embryo in a larger gene space, we performed a principal component analyses (PCA) using expressed genes [FPKM>1 in at least 50% of cells within a lineage assigned in the Xiang et al. (2020) study] with high variability (CV >0.5) across all 555 cells. Color coding cells with the lineages assigned in the Xiang et al. study reveals that the first principal component separates trophectoderm, primitive endoderm and epiblast cell types while the second principal component separates the ‘AME, ‘intermediate and primitive streak cells from the rest. Restricting the PCA to more variable genes (CV>1, CV>1.5) puts most of the ‘AME, ‘intermediate and primitive streak cells together on PC1, distinct from the rest of the cells (Fig. 3A). This clustering result is broadly similar to the one shown in Fig. 2B of Xiang et al. (2020), where the PSA cluster in Fig. 2B places D14 ‘AME, ‘intermediate and primitive streak cells together, distinct from the rest of the cells.

To determine the gene categories (Ensembl biotypes) that contribute the most to the two principal components in the above analyses, we plotted the normalized PC coefficient of different gene categories for each principal component. The top two contributors are protein coding genes and pseudogenes. Strikingly, the contribution of pseudogenes increases as the ‘AME-‘intermediate-primitive streak cluster moves to a distinct PC1 (Fig. 3B). This cluster of cells also express a higher fraction of pseudogenes than the other cell types (Fig. S3).

Pseudogenes are homologous to protein coding genes but with a frameshift or stop codon, which renders them non-translational (Pink et al., 2011). While there is some speculation on the role of pseudogenes in gene regulation (Milligan and Lipovich, 2015), there is no conclusive evidence for an essential role of pseudogenes in early mammalian development. Hence, we repeated the PCA with only protein coding genes under the same gene selection criteria as before. Contrary to the previous analyses, the ‘AME are now distinct from the ‘intermediate and primitive streak cells (Fig. 3C). Instead, most ‘AME cells now (10/13 in CV>0.5, >1.0; 7/13 in CV>1.5) share PC1 with trophoblast cells. Plotting the D12 and D14 data separately shows that even with the most restricted gene set (CV>1.5), most (7/11) D14 ‘AME cells share PC1 with trophoblast cells (Fig. 3D). This indicates that transcriptional similarity of protein coding genes is very high between ‘AME and trophoblast cells, consistent with previous section, and their placement with primitive streak cells in the analyses of Xiang et al. is due to similar expression of pseudogenes.

### ‘AME contain a mix of EVT, STB and ambiguous cells

**Fig. 4.**
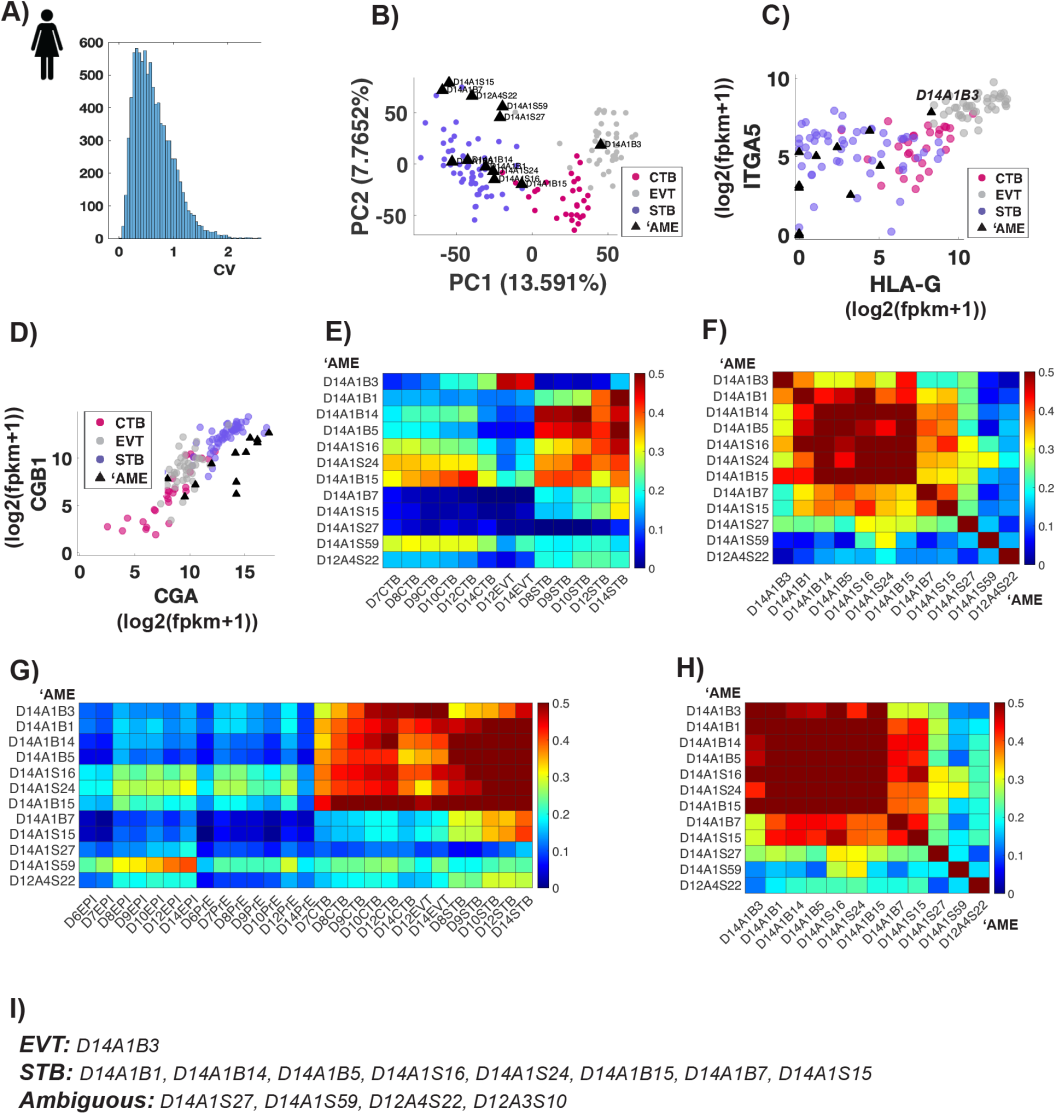
**‘AME cells contain a mix of EVT, STB and ambiguous cells.** (A) Histogram of coefficient of variation of expressed genes in D12, D14 CTB, EVT, STB and ‘AME cells. (B) PCA of variable genes (CV>0.5) across cells in D12, D14 CTB, EVT, STB and ‘AME cells*.* Methodology and axis labels same as in Fig. 3. (C,D) Scatter plots showing expression of indicated genes in D12,14 CTB, EVT, STB and ‘AME cells. (E,G) Heatmap showing Pearson correlation coefficients for expression of variable genes [E: genes used for PCA in Fig. 4B; G: genes used for PCA in Fig. 3C (CV 0.5)] between individual ‘AME cells and indicated cell type averages. (F,H) Heatmap showing Pearson correlation coefficients for expression of variable genes [F: genes used for PCA in Fig. 4B; H: genes used for PCA in Fig. 3C (CV 0.5)] between individual ‘AME cells. (I) ‘AME cells reclassified as EVT, STB and ambiguous cells.

To further determine which trophoblast cell type ‘AME corresponds to, we repeated the principal component analyses using expressed genes with high variability (CV>0.5; 5296 genes) between D12,14 amnion, CTB, STB and EVT cells (Fig. 4A). We removed one ‘AME cell (D12A3S10) from this analysis, as it exhibits high correlation of lineage specific gene expression with epiblast cells and is placed either in the primitive endoderm or epiblast cluster in all PCA plots (Figs 1A and 3D).

PCA reveals three distinct populations of ‘AME (Fig. 4B). One cell is placed with EVT cells whereas the others are divided into two groups, both of which comprise STB cells. These two groups might represent different stages within STB maturation. The ‘AME cell in the EVT cluster expresses known EVT markers – HLA-G and ITGA5 (Okae et al., 2018; Knöfler et al., 2019), whereas other ‘AME cells do not (Fig. 4C). All ‘AME cells express high levels of human chorionic gonadotrophins genes (hCGA, hCGB1), comparable to STB cells (Fig. 4D; Okae et al., 2018; Knöfler et al., 2019). Based on hCG protein staining in Extended Data figure 4U of Xiang et al. (2020), they argue that amnion cells express hCG protein, however, the data shown in that figure is unclear. The cells that have high hCG lie outside a layer of cells surrounding the amniotic cavity, and likely represent STB cells. To distinguish between the two cell types, it is necessary to show an overlap with other known markers. Moreover, hCG is a secreted protein, so its presence near a cell need not imply production in the same cell. Thus, hCG immunostaining alone is not a good indication that it is expressed by amnion cells.

Consistent with PCA, a correlation analyses of expression levels in same gene set also reveals three distinct populations of ‘AME. One cell (1/12) is transcriptionally correlated (Pearson correlation coefficient >0.4) with EVT cells, half of the cells (6/12) are correlated with STB cells, and the rest (5/12) show either low or no correlation with any of three trophoblast cell types (Fig. 4E). To determine if this third population (5/12) comprises a distinct cell type, we examined the pairwise correlation for gene expression of the same gene set within individual ‘AME cells (Fig. 4F). The heterogeneity within these five cells, highlighted by low cell-to-cell correlation values, argues against this. Repeating the correlation analyses for a larger gene set (CV>0.5 across all cells), reveals that most ‘AME cells (9/12) are transcriptionally correlated with one or more trophoblast lineages, but three cells (D14A1S27, D14A1S59, D12A4S22) still remain transcriptionally distant (Fig. 4G,H). Within the known lineage marker gene space, D14A1S27, D14A1S59, D12A4S22 correlate with CTB/EPI, CTB and PrE cells respectively but in a larger gene space the lineage relationship is lost (Figs 1A and 4E–H). Due to this apparent contradiction, we cannot conclusively determine an identity for these three cells. Amongst the rest, we classify 8/9 cells as mislabeled STB and 1 cell as mislabeled EVT cell.

Taken together, our results suggest that the data in Xiang et al. (2020) are not sufficient to determine the transcriptome of human amnion.

### BMP-treated hESCs are transcriptionally more similar to early post-implantation monkey amnion than monkey trophectoderm

In the absence of a unique human amnion transcriptome, we turned to the post implantation monkey embryo to resolve the identity of BMP-treated hESCs. We have previously shown that sparsely seeded hESCs treated with BMP4 ligands for 42 h transcriptionally resemble trophectoderm cells, and not extra-embryonic mesoderm cells (Chhabra et al., 2019). In this section, we revisited that data and compared the transcriptional similarity of BMP-treated hESCs with monkey amnion and trophectoderm lineages.

We first defined a set of lineage specific genes for the early post implantation monkey amnion, trophectoderm and epiblast (D11-14), and then compared the expression of those genes in monkey embryo with BMP-treated hESCs. We chose early stages (D11-14) of monkey post implantation development because the transcriptional similarity with corresponding human stages is higher at these stages compared to the later (D16–17) (Fig. 2A).

**Fig. 5.**
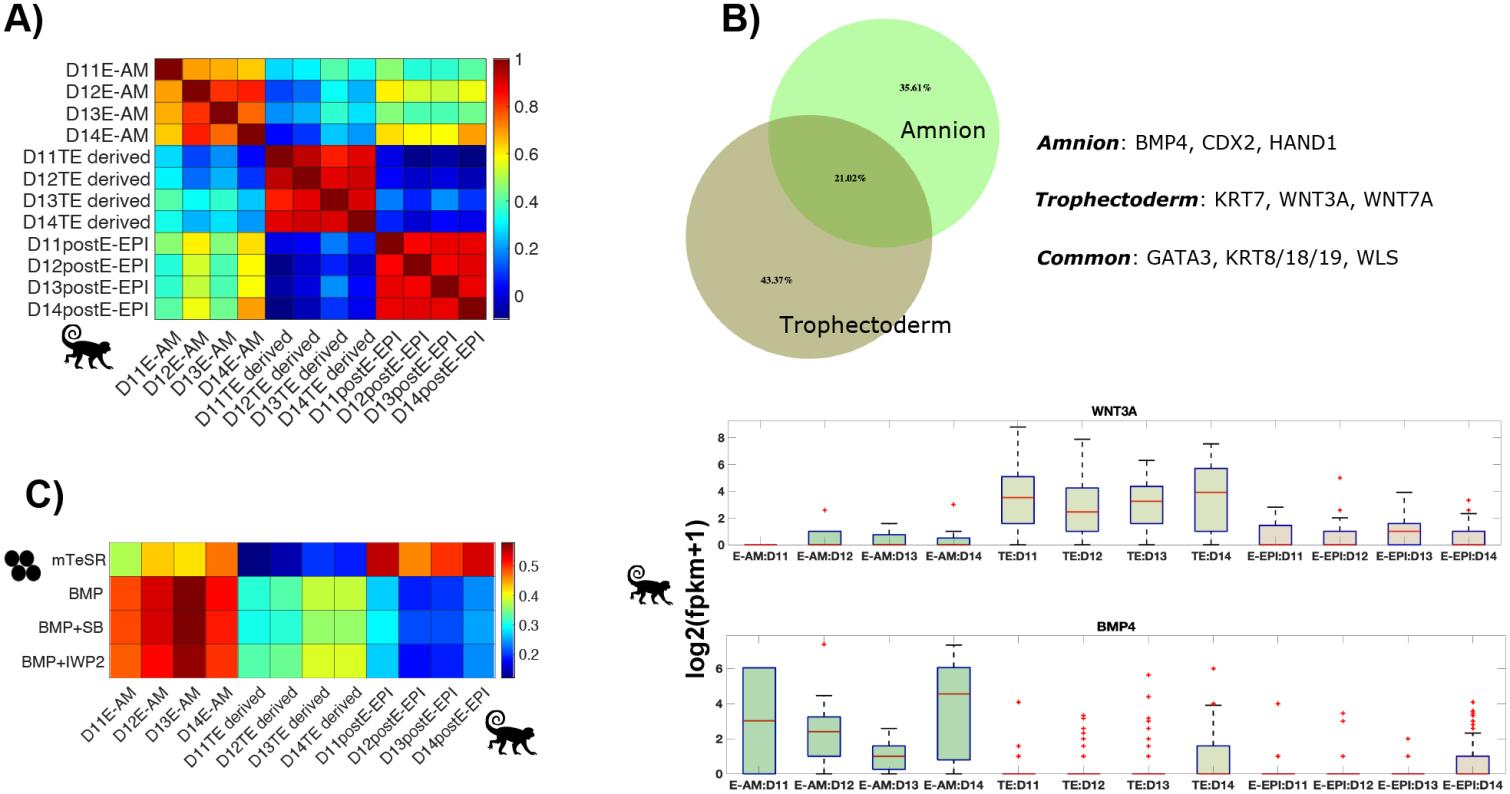
**BMP-treated hESCs transcriptionally resemble monkey early post-implantation amnion cells.** (A) Pearson correlation coefficients between indicated samples for 571 lineage-specific genes determined from *in vivo* monkey embryo data. (B) Venn diagram for differentially upregulated genes in indicated samples compared to the epiblast. Amnion refers to samples labeled as E-AM, trophectoderm to TE derived and epiblast to postE-epiblast in A. (C) Pearson correlation coefficients between indicated samples for 560 lineage-specific genes determined from *in vivo* monkey embryo data.

To determine lineage-specific genes, we extracted genes that are differentially expressed between that lineage and at least one of the other two lineages [fold change >5, false discovery rate (FDR)=0.01]. From this list, we excluded genes that are differentially expressed between different time points within that lineage [embryonic day (D)11–14] to reduce noise within the lineage and further removed genes with a low expression value [fragments per kilobase of transcript per million mapped reads (FPKM)<5 in at least two of the four time points for that lineage]. This gave a list of 571 lineage-specific genes (Table S1). These genes clearly separate the three lineages transcriptionally (Fig. 5A). Examining the genes differentially upregulated in the amnion and trophectoderm compared to the epiblast reveals that the monkey amnion differentially upregulates BMP4, whereas the trophectoderm differentially upregulates WNT3A, consistent with their *in-situ* expression (Fig. 5B; Sasaki et al., 2016).

Finally, comparing the expression of lineage-specific genes in BMP-treated hESCs and monkey embryo revealed that BMP-treated hESCs are transcriptionally more similar to monkey amnion than monkey trophectoderm-derived cells (Fig. 5C). This is intriguing because previous studies have shown that BMP-treated hESCs’ can differentiate towards mature trophectoderm subtypes (Xu et al., 2002). Assuming transcriptional similarity between human and monkey amnion, this result suggests that hESCs may possess a remarkable ability to differentiate into mature trophectoderm cells via an amnion-like transcriptional state.

### Xiang et al. dataset contains additional mislabeled cellular populations

Correlation analyses of human lineage specific genes across different cell types in the Xiang et al. dataset revealed that two additional cell populations – ‘intermediate cells and inner cell mass (‘ICM) cells, are likely mislabeled (Fig. 1E,F).

#### ‘Intermediate cells are mislabeled extra embryonic mesoderm cells

‘Intermediate cells are a novel cell type identified in the Xiang et al. study. In the tSNE analyses presented in figure 2B in Xiang et al., D12 ‘intermediate cells are placed with epiblast and amnion cell types whereas most D14 ‘intermediate cells are placed in the PSA cluster with amnion and primitive streak cell types. This indicates that ‘intermediate cells represent an epiblast-derived cell population.

**Fig. 6.**
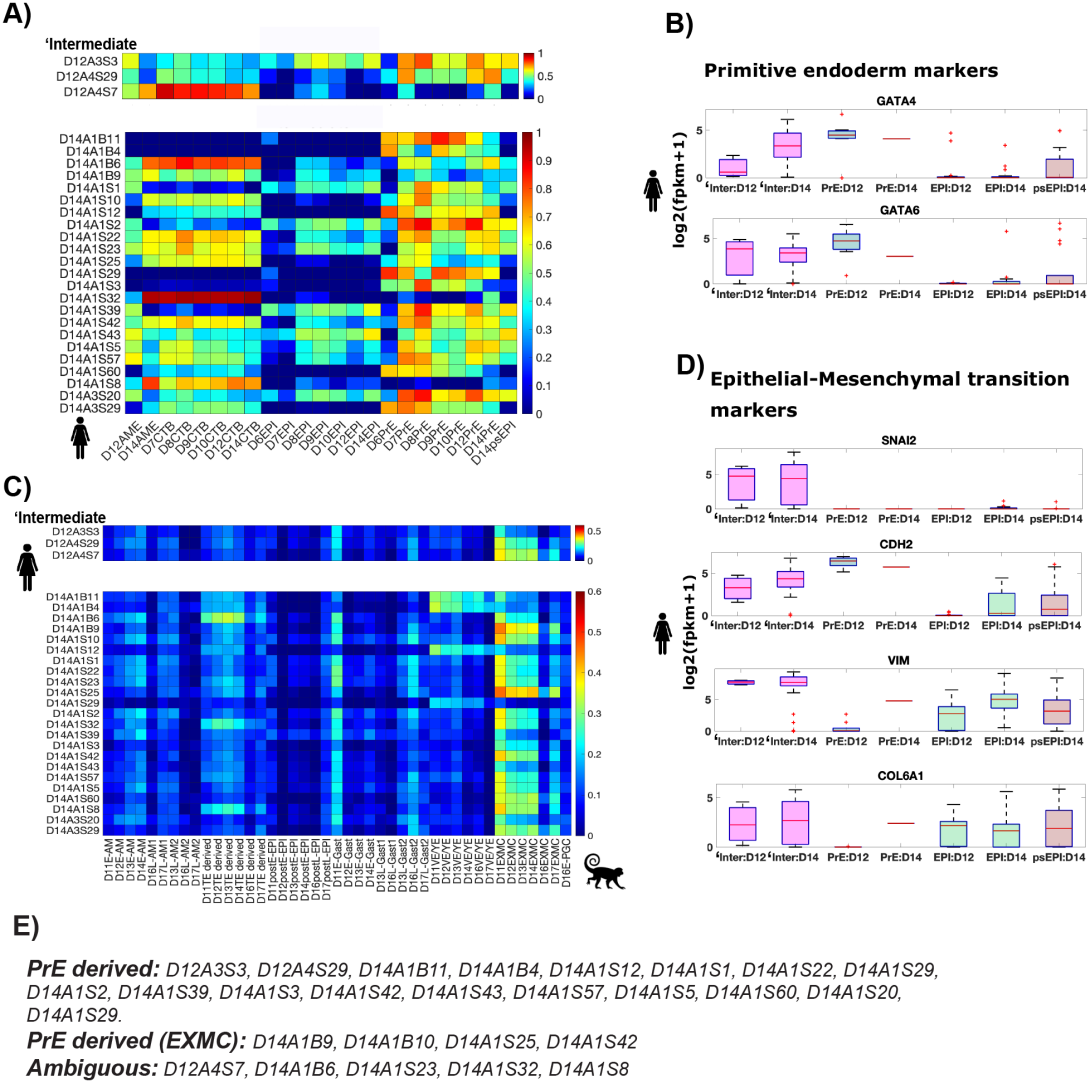
**‘Intermediate cells are mislabeled extra embryonic mesoderm cells.** (A,C) Heatmap showing Pearson correlation coefficients for expression of known lineage markers in human embryo (A) or variable genes in the monkey embryo (C) between individual ‘intermediate cells and indicated cell type averages. Genes in C are same as those in Fig. 2D. (B,D) Box plots showing expression of indicated genes in indicated lineages.

However, in the lineage-specific gene space in our analyses, ‘intermediate cells exhibit maximum transcriptional similarity with primitive endoderm cells (Fig. 1E,F), not epiblast cells. This trend is preserved at the level of single cells, where a majority of ‘Intermediate cells are not transcriptionally correlated with the epiblast or epiblast-derived primitive streak cells (Fig. 6A). Consistent with this, most of the ‘intermediate cells do not express pluripotency and primitive streak markers, thus questioning their placement in the PSA cluster (Fig. S4A,B).

Most ‘intermediate cells (20/26; 2/3 D12, 18/23 D14) are transcriptionally correlated with primitive endoderm (PrE) cells (Fig. 6A). Consistent with this, intermediate cells express primitive endoderm markers like GATA4/6 at a level comparable to primitive endoderm cells on the same day (Fig. 6B). This suggests that ‘intermediate cells represent mislabeled primitive endoderm cells or primitive endoderm derived cells.

Similar to ‘AME, the misclassification of ‘intermediate cells is also due to the inclusion of pseudogenes. When the principal component analysis is limited to protein coding genes, ‘intermediate cells share PC1 with PrE cells on both D12 and D14 in all the three gene sets analyzed (Fig. 3A,C,D), indicating a high transcriptional similarity of protein coding genes between ‘intermediate cells and PrE cells.

In the variable gene space of the monkey embryo that separates the two primitive endoderm derived lineages – the EXMC and the VE/YE, most ‘intermediate cells (22/26; 3/3 D12, 19/23 D14) exhibit maximum transcriptional similarity with monkey EXMC cells. Consistent with this, ‘intermediate cells express known monkey EXMC genes like GATA4, GATA6, COL6A1, VIM, CDH2, SNAI2 (Fig. 6B,D; Nakamura et al., 2016, extended figure 5D).

We relabel ‘intermediate cells as primitive endoderm derived if they exhibit maximum correlation with PrE lineage in the human embryo (condition1) (Fig. 6A), and further classify them as EXMC cells if they exhibit a high correlation with monkey EXMC (correlation coefficient >0.4) and satisfy condition 1. The cells that do not satisfy condition 1 are labelled as ambiguous cells because they are transcriptionally correlated with CTB/EPI cells but cluster with PrE cells in the PC space (Figs 3C and 6E). It is worth noting that the images in Xiang et al. (2020) do not show a distinct EXMC cell population. However, it is plausible that some primitive endoderm cells have started differentiating towards the EXMC, but a separate EXMC tissue is not yet formed.

#### ‘ICM cells are mislabeled CTB cells

During implantation, the epiblast transitions between the naïve and primed pluripotent states, in both mouse and monkey. At the molecular level, this transition results in a reduced expression of naïve pluripotency genes, along with a sustained expression of core pluripotency genes (Nichols and Smith, 2009; Nakamura et al., 2016). As precursors of epiblast cells, ICM cells are expected to be transcriptionally similar to epiblast cells and express either higher or comparable levels of naïve pluripotency markers as pre-implantation epiblast cells. However, the ‘ICM cells identified in Xiang et al. (2020) do not satisfy these conditions.

**Fig. 7.**
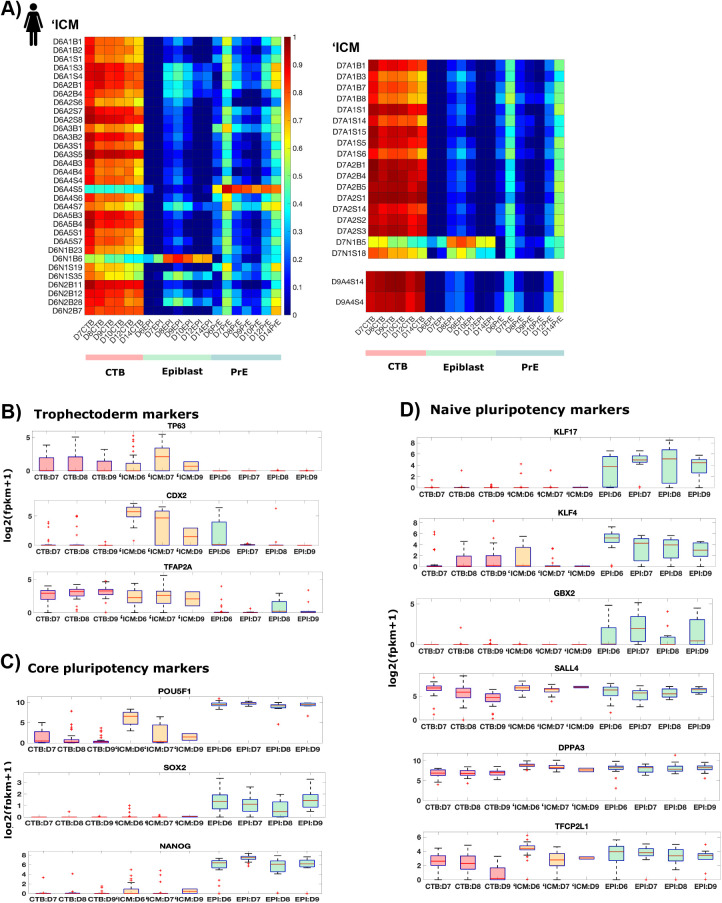
**‘ICM cells are mislabeled CTB cells.** (A) Heatmap showing Pearson correlation coefficients for expression of known lineage markers between individual ‘ICM cells and indicated cell type averages. (B–D) Box plots showing expression of indicated genes in indicated lineages.

Comparing expression of lineage specific genes in individual ‘ICM cells with average expression of those genes in different lineages in the embryo reveals that a majority of these cells (49/52) are transcriptionally correlated with CTB, not epiblast cells (Fig. 7A). Consistent with this, ‘ICM cells express other known trophoblast markers – TP63, TFAP2A, CDX2 at a level comparable with CTB cells on the same days (Fig. 7B). On the other hand, these cells do not express core pluripotency markers – NANOG, SOX2 and OCT4 (Fig. 7C). These data contradict previous literature that shows that D6-9 ICM cells express pluripotency, not trophectoderm genes (Roode et al., 2012; Niakan and Eggan, 2013; Blakeley et al., 2015; Deglincerti et al., 2016; Shahbazi et al., 2016).

Xiang et al. (2020) state that they observe a gradual maturation of the epiblast from the naïve to the primed pluripotency state (Xiang et al., 2020, extended figure 9). However, naïve pluripotency markers – KLF17, KLF4, GBX2 – are expressed in fewer ‘ICM cells and at a lower level, compared with epiblast cells. Other naïve pluripotency markers, SALL4 and DPPA3, are expressed in comparable levels in ‘ICM, epiblast and CTB cells, indicating that these markers are not specific to ICM/epiblast. The only exception is TFCP2L1, which exhibits a slightly higher median expression in day 6 ‘ICM cells, compared to epiblast and CTB cells (Fig. 7D). However, TFCP2L1 protein is expressed at comparable levels in both CTB and ‘ICM cells of the day 6 human embryos (Xiang et al., 2020, extended data figure 9a), indicating that TFCP2L1 is also not specific to ICM/epiblast. Taken together, this data shows that the cells labelled as ‘ICM express naïve pluripotency markers at a level comparable to CTB cells, and lower than epiblast cells.

In D6-9 human embryos, the absolute number of trophectoderm cells is higher than ICM/epiblast cells (Roode et al., 2012; Niakan and Eggan, 2013; Blakeley et al., 2015; Deglincerti et al., 2016; Shahbazi et al., 2016). Thus, it is surprising to obtain 32 ‘ICM cells, 28 epiblast cells and 0 CTB in D6 human embryos (Fig. 1A). It is more likely that 30/32 ICM cells, which exhibit transcriptional similarity with CTB cells, are mislabeled CTB cells (Fig. 7A).

It is worth noting that the cells of the D5 human embryo cannot be distinguished based on the known lineage markers as they co-express markers of the three lineages (Stirparo et al., 2018). However, most of the putative ICM cells in this dataset (49/52) clearly correlate more with trophoblast cells than the other two lineages and express trophoblast markers on a par with CTB cells, indicating that they do not correspond to an early heterogenous population and are likely mislabeled CTB cells.

Taken together, the above analyses suggest that 49/52 ICM cells – 30/32 D6, 17/18 D7 and 2/2 D9 ICM cells are mislabeled CTB cells on the corresponding days. Of the remaining 3 cells, 2 represent epiblast cells (D6N1B6, D7N1B5) and 1 represents primitive endoderm (D6A4S6) on the corresponding day, as indicated by the transcriptional similarity of known lineage genes (Fig. 7A).

## DISCUSSION

hESCs offer a unique opportunity to probe early stages of human development. Their immense potential to differentiate into a variety of different cell types offers a valuable resource for both translational and fundamental research. However, to make accurate inferences, it is essential to determine the identity of cells obtained by differentiation of hESCs through careful comparisons with embryos.

In this study, we examined the transcriptome of the much-debated BMP-treated hESCs, which have been variably considered similar to three extra-embryonic cell types – the trophectoderm, the extra-embryonic mesoderm and the amnion (Xu et al., 2002; Bernardo et al., 2011; Shao et al., 2017a,b). Comparing the transcriptome of these cells with peri-implantation monkey embryos shows that they are more similar to monkey amnion cells than trophectoderm or extra-embryonic mesoderm cell types (Fig. 5). Together with a wealth of previous results that have shown the ability of BMP-treated hESCs to differentiate into mature subtypes, this result indicates that hESCs might possess a unique ability to differentiate into trophectoderm cells via an amnion-like intermediate transcriptional state. Below, we elaborate previous results supporting this argument.

### Trophectoderm, extra-embryonic mesoderm or amnion cells?

Comparison of hESC derived lineages to mouse development argues against a trophectoderm identification: The expression of mature trophectoderm markers by BMP-treated hESCs sparked off a debate on the physiological relevance of these cells as it contradicted previous results in the mouse literature (Xu et al., 2002). Mouse embryonic stem cells (mESCs) transplanted into mouse embryos rarely contribute to trophectoderm lineages (Beddington and Robertson, 1989) and cannot be differentiated to trophectoderm cell types without genetic perturbations (Niwa et al., 2000). This is consistent with the fact that mESCs are derived from the blastocyst of pre-implantation embryos after the trophectoderm lineage has already segregated, and consequently lack the potential to differentiate into trophectoderm subtypes (Nichols and Smith, 2012). As their human homologs, hESCs were expected to similarly lack this potential but a wealth of data suggests that this might not be true.

#### Evidence for trophectoderm cell fate

In 2002, a study showed that hESCs treated with BMP4 for a week differentiate into a cells expressing common trophectoderm markers like TFAP2A/C, GATA2/3 (Ma et al., 1997; Richardson et al., 2000; Xu et al., 2002; Bai et al., 2012), syncytiotrophoblast markers like CG-A, CG-B (Muyan and Boime, 1997; Lacroix et al., 2002) and extra-villous trophoblast markers like HLA-G1 (Ferreira et al., 2017) to varying degrees at different time points over the course of differentiation. The formation of syncytiotrophoblast cells was further supported by a similar morphology and hormonal profile as syncytiotrophoblast cells *in vivo*. That is, under these conditions, hESCs differentiated into large multi-nucleated cells and secreted placental hormones including hCG, estradiol and progesterone (Xu et al., 2002). Since then, these findings have been replicated in other labs and protocols have been refined to obtain near pure populations of different trophectoderm subtypes starting from BMP-treated hESCs (Das et al., 2007; Aghajanova et al., 2012; Amita et al., 2013; Horii et al., 2016). Studies have also elucidated that the differentiation of hESCs to mature trophectoderm subtypes occurs via TP63 positive cytotrophoblast-like progenitor state similar to their *in vivo* developmental path (Li et al., 2013), consistent with our previous findings showing transcriptional similarity between hESCs treated with BMP4 for 2 days and trophectoderm cells of day 7 human embryos, which are presumably cytotrophoblast cells (Chhabra et al., 2019).

#### Evidence for extra-embryonic mesoderm cell fate

In 2011, another study proposed that BMP4 treated hESCs expressing trophoblast genes like CDX2 and KRT7 represent extra-embryonic mesoderm cells, which have been shown to express these markers in the mouse embryo (Bernardo et al., 2011). This seemed to resolve the debate because mouse extra-embryonic mesoderm cells are derived from epiblast-derived primitive streak and thus likely reflect a natural developmental path of embryonic stem cells (Kinder et al., 1999). However, there are problems with this conclusion. First, the culture conditions for trophoblast differentiation in this study were optimized for mouse epiblast cells not hESCs, and consequently the differentiated cells passed through a BRA/CDX2 double positive state, which is not observed in other BMP-treated hESCs (Chhabra et al., 2019). Second, the extra-embryonic mesoderm in humans and monkeys is present prior to primitive streak formation and exhibits transcriptional similarity with primitive-endoderm cells, indicating a different developmental origin than mouse extra-embryonic mesoderm (Luckett, 1978; Enders and King, 1988; Nakamura et al., 2016; Ross and Boroviak, 2020). BMP-treated hESCs do not express key primitive-endoderm markers like GATA4 and GATA6, and thus, are unlikely to represent extra-embryonic mesoderm cells (Chhabra et al., 2019).

#### Evidence for amnion cell fate

In 2016, a new *in vitro* model of human amniogenesis was proposed where hESCs embedded in a soft gel bed in 3D and grown in media supplemented with matrigrel differentiate to form squamous epithelia expressing known trophoblast and amnion markers in a BMP dependent manner (Shao et al., 2017a,b). Although the authors of the study label these cells as amnion, they express many known trophoblast genes like TP63, CDX2, GATA2/3, TFAP2C. As the amnion cells are derived from the epiblast in humans (Dobreva et al., 2010), amnion differentiation represents a natural developmental path of hESCs and thus potentially resolves the debated identity of BMP-treated hESCs. But monkey amnion cells also express some trophoblast genes (Sasaki et al., 2016), indicating transcriptional similarity between the two lineages. Determining if this similarity extends to the entire transcriptome or if there are genes specific to only one lineage, and if BMP-treated hESCs transcriptionally correlate with one lineage more than the other required a higher resolution transcriptional profiling of the two lineages, which was not available until recently (Ma et al., 2019; Niu et al., 2019). Using one of these published datasets (Ma et al., 2019), we determined the extent of transcriptome similarity between early monkey amnion, trophoblasts and BMP-treated hESCs. Our analyses reveal that BMP-treated hESCs are transcriptionally more similar to monkey early amnion cells than trophectoderm cells (Fig. 5C).

Taken together, the results reveal two facts – (1) BMP-treated hESCs form cells that morphologically and molecularly resemble mature trophoblasts, as argued earlier. (2) BMP-treated hESCs are transcriptionally more similar to monkey amnion cells than monkey trophectoderm cells. Assuming transcriptional similarity between human and monkey amnion and trophoblast lineages at this stage of development, it can be reasoned that BMP-treated hESCs possess a unique ability to differentiate into mature trophectoderm cell types via an amnion-like transcriptional state.

A recently published preprint also comes to the same conclusion, although from a different starting point. The authors of the study show hESCs treated with a chemical cocktail devoid of BMP, activate endogenous BMP signaling and differentiate into mature trophectoderm subtypes via an amnion-like intermediate state (Ohgushi and Eiraku, 2021preprint). This indicates that BMP signaling, whether provided exogenously or activated endogenously, drives hESCs towards the trophectoderm path via an amnion-like intermediate. Whether this developmental path is taken by trophoblast cells *in vivo* and whether the transcriptional similarity translates into functional plasticity between the two lineages where amnion cells can differentiate into trophoblast cell types and vice versa remains to be experimentally tested.

### Pseudogenes and mislabeled cell types

Our analyses also revealed that the inclusion of pseudogenes leads to the mislabeling of amnion cells in the Xiang et al. dataset (Xiang et al., 2020). In the absence of pseudogenes, ‘AME cells do not form a separate cluster in the principal component analyses and are instead spread across the principal component space, with most of the cells in the trophoblast region (Fig. 3C). Restricting the analyses to amnion and trophectoderm cell types further revealed that most of the ‘AME cells transcriptionally resemble syncytiotrophoblast cells (Fig. 4). The erroneous results obtained when pseudogene expression is not excluded highlight the need to carefully compile appropriate lists of genes to compare different cell populations. A general method for doing so is an important topic for future study.

We found additional mislabeled cellular populations in the Xiang et al. dataset. One of these is the ‘intermediate cell population. Although Xiang et al. do not comment on their *in vivo* identity, their placement with the epiblast and amnion cells implies that they presumably represent an epiblast derived cell population. However, we show that excluding pseudogenes changes their position in the principal component space and moves them closer to the primitive endoderm cluster, indicating that they likely represent primitive-endoderm derived cells (Fig. 3C). Comparison with the monkey embryo revealed that some of these cells likely represent extra-embryonic mesoderm cells which are known to express key primitive endoderm genes (Fig. 6C; Nakamura et al., 2016). It is worth noting that there is no morphological extra-embryonic region covering the amnion-embryo-primitive endoderm region in the Xiang et al. study (Xiang et al., 2020). However, it is plausible that some cells have started differentiating towards the extra-embryonic mesoderm but have not occupied their morphological location yet. In the future, time lapse imaging studies could discern the precise dynamics of extra-embryonic mesoderm specification and migration.

## MATERIALS AND METHODS

All the analyses were performed at the level of genes. For genes with multiple transcripts, cumulative expression of all transcripts was considered as the gene read count and the ensembl gene id of most expressed transcript was considered as its gene id. PCA and correlation analyses within a given dataset was performed on log transformed read counts [log2 (FPKM+1)]. For all analyses except in Fig. 5, genes were selected based on expression counts (FPKM>1 in at least 50% cells of a given lineage) and variability across cells (CV threshold). To determine lineage specific genes in Fig. 5, we used EBSeq with an FDR cutoff of 0.01 for pairwise differential gene analyses in order to reduce the overlap of lineage-specific genes across lineages (Leng et al., 2013). We further filtered this set to exclude lowly expressed (FPKM<5 in more than two samples of a given lineage) and lowly upregulated [fold change (realFC parameter in ebseq output file) <5] genes. CV thresholds and number of genes are indicated in relevant figures and figure legends. For analyses between the two datasets, each dataset was filtered to exclude non-expressed genes (FPKM=0 in all cells within a dataset), after which the log normalized read counts [log2(FPKM+1)] were transformed into z scores. Only genes retained in both datasets after filtering were utilized for correlation analyses. Human gene orthologs of monkey genes were obtained from the supplementary information in Nakamura et al. (2016). Analysis code can be found on Github: https://github.com/warmflashlab/Chhabra2021.

## Supplementary Material

Supplementary information
