# Supplementary material for: BMP-treated human embryonic stem cells transcriptionally resemble amnion cells in the monkey embryo
Source: Biol Open. 2021 Sep 22;10(9):bio058617. doi: 10.1242/bio.058617 (PMC8502258; doi:10.1242/bio.058617)
Supplement: Supplementary information [file biolopen-10-058617-s1.pdf]

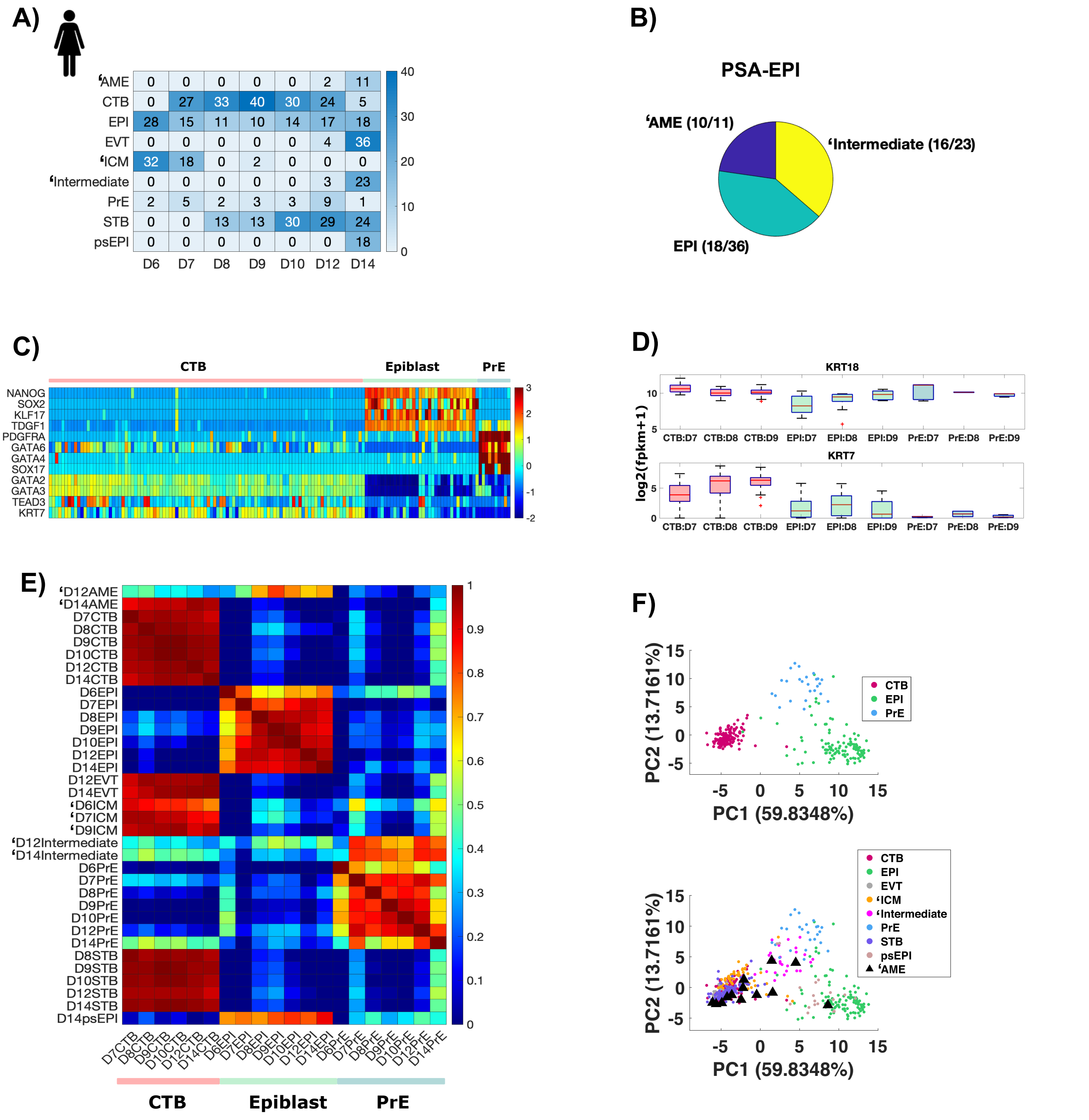

Fig. S1. Known lineage markers separate human epiblast, trophectoderm and primitive endoderm lineages.

(A) No. of cells corresponding to each cell type on each day. psEPI cells are epiblast cells in the primitive streak anlage cluster. *(B)* Distribution of cells in primitive streak anlage (PSA) cluster. Data in A and B is based on information in Supplementary table 8 (S8.1, S8.3) in Xiang et al 2019 study. *(C)* Heatmap showing expression of the 12 known lineage markers in each cell in D7-9 cytotrophoblast (CTB), epiblast (EPI) and primitive endoderm (PrE) lineages. Values correspond to z-scores of indicated genes. Z- scores were calculated for each gene across all cells in D7-9 EPI, PrE and CTB lineages. *(D)* Box plots showing expression of indicated genes in indicated lineages. *(E)* Heatmap showing Pearson correlation coefficients of average expression of known lineage markers in each cell type. *(F)* PCA of known lineage marker gene expression across all 555 cells. In the top plot, a subset of cells corresponding to CTB, EPI and PrE lineages are shown.

Biology Open • Supplementary information

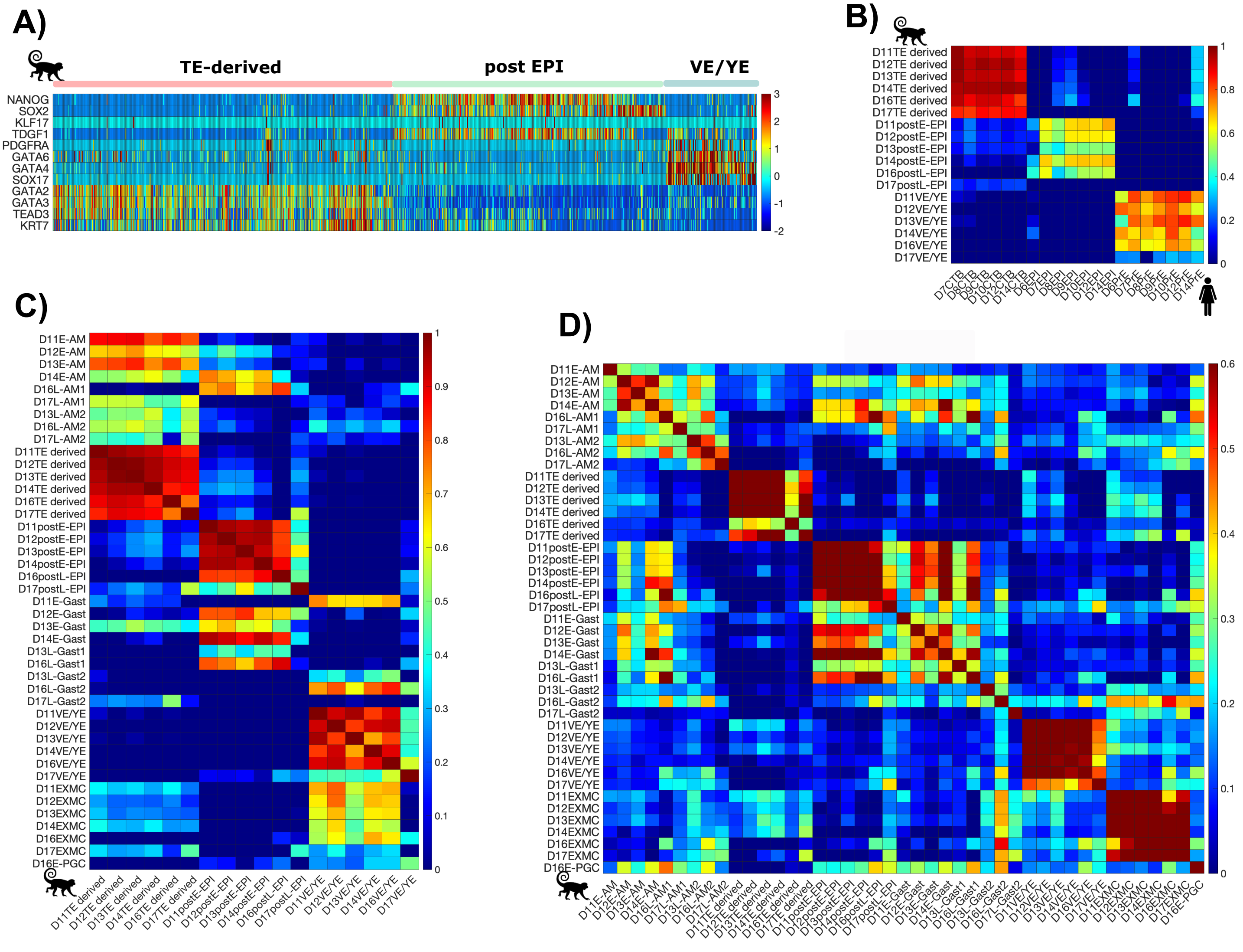

Fig. S2. Human lineage specific genes delineate three primary lineages of the monkey embryo but do not distinguish amnion and trophectoderm lineages

(A) Heatmap showing expression of human known lineage markers (same genes as in Fig1C) in each cell in D11-D17 trophectoderm-derived, post implantation epiblast and visceral endoderm/yolk-sac endoderm (VE/YE) lineages in the monkey embryo. Values correspond to z- scores of indicated genes. Z- scores were calculated for each gene across all cells in the plotted lineages. *(B-D)* Heatmap showing Pearson correlation coefficients of average expression of either known lineage markers (B, C) or variable genes in the monkey embryo ((D); CV>1 across 1453 monkey cells; 2440 genes) in indicated cell types.

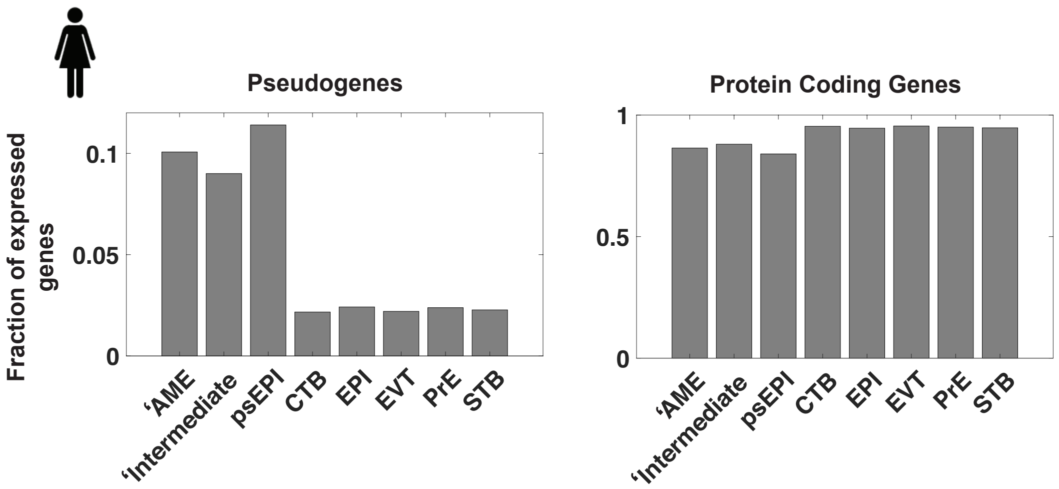

Fig. S3. 'AME, Intermediate and psEPI cells express a higher fraction of pseudogenes than other cell types

Fraction of expressed genes (FPKM>1 in at least 50% cells of a given lineage) that correspond to pseudogenes and protein coding genes in D12 and D14 samples of indicated cell types.

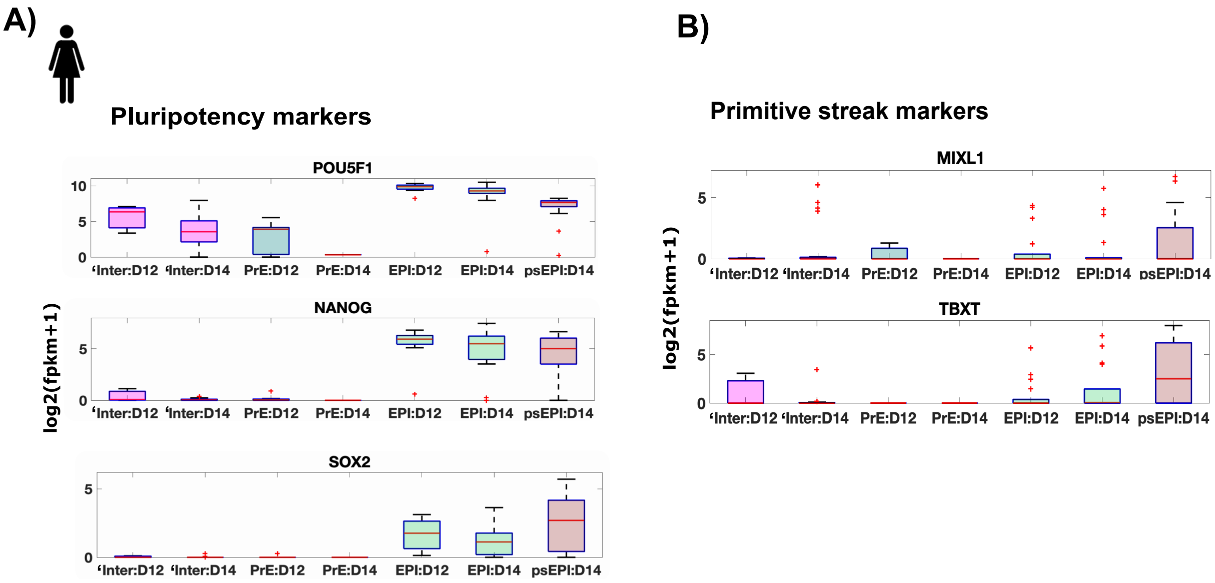

Fig. S4. 'Intermediate cells do not express pluripotency and primitive streak markers *(A, B))* Box plots showing expression of indicated genes in indicated lineages.

Table S1.

Click here to download Table S1

Biology Open • Supplementary information
